# Anti-BCMA CAR-T Cell Therapy in Relapsed/Refractory Multiple Myeloma Patients With Extramedullary Disease: A Single Center Analysis of Two Clinical Trials

**DOI:** 10.3389/fimmu.2021.755866

**Published:** 2021-10-29

**Authors:** Yimei Que, Menglei Xu, Yanjie Xu, Varlene Daniela Fernandes Almeida, Li Zhu, Zhiqiong Wang, Ying Wang, Xian Liu, Lijun Jiang, Di Wang, Chunrui Li, Jianfeng Zhou

**Affiliations:** ^1^ Department of Hematology, Tongji Hospital, Tongji Medical College, Huazhong University of Science and Technology, Wuhan, China; ^2^ Tongji Medical College, Huazhong University of Science and Technology, Wuhan, China; ^3^ Immunotherapy Research Center for Hematologic Diseases of Hubei Province, Wuhan, China

**Keywords:** BCMA (TNFRSF17), car-t, relapsed, refractory, extramedullary, multiple myeloma

## Abstract

**Background:**

The prognosis of relapsed/refractory multiple myeloma (RRMM) patients with the extramedullary disease was significantly poor. Extramedullary multiple myeloma (EMM) patients gained limited benefits from traditional drugs. Anti-B cell maturation antigen (BCMA) chimeric antigen receptor (CAR) T-cell therapy seems to be a promising approach to treat RRMM patients. However, very few clinical studies are designed for EMM. Our study aimed to compare and assess the safety, efficacy, and pharmacokinetics of anti-BCMA CAR-T cell therapy in EMM and non-EMM.

**Methods:**

The results from published anti-BCMA CAR-T clinical trials, in which raw data of EMM patients were available, were reviewed and summarized. Two trials conducted in our clinical centers were analyzed and presented with detailed data.

**Results:**

According to published anti-BCMA CAR-T clinical trials, the ORR of EMM ranged from 57% to 100%, with the complete remission (CR) rate of 29% to 60%. Between February 22, 2017, and September 26, 2019, a total of 61 subjects (EMM 25; non-EMM 36) received anti-BCMA CAR-T cell infusion. The data-cutoff date was April 1, 2021. There were no statistical differences between EMM and non-EMM groups in adverse events (AEs), including cytokine release syndrome (CRS). The most common AEs of grade ≥ 3 in both groups were hematologic toxicities. There was no significant difference in the objective response rate (ORR) and ≥ complete remission (CR) rate between both groups. However, the ≥ CR rate of the EMM group was lower than the non-EMM group receiving the fully human anti-BCMA CAR-T cell therapy (*p* = 0.026). The median progression-free survival (PFS) for EMM and the non-EMM group was 121 days and 361 days, respectively (*p* = 0.001). The median overall survival (OS) for EMM and the non-EMM group was 248 days and 1024 days, respectively (*p* = 0.005). The C_max_ and AUC_0-28d_ for EMM group were lower than non-EMM group (C_max_, *p* = 0.016; AUC_0-28d_, *p* = 0.016). Extramedullary disease was an independent prognostic risk factor for PFS (hazard ratio, 2.576; 95% CI, 1.343 to 4.941; *p* = 0.004) and OS (hazard ratio, 2.312; 95% CI, 1.165 to 4.592; *p* = 0.017) in RRMM patients receiving anti-BCMA CAR-T cell therapy.

**Conclusions:**

Based on our results, EMM patients could benefit from the two anti-BCMA CAR products, although they had a shorter PFS and OS compared with non-EMM patients.

**Clinical Trial Registration:**

http://www.chictr.org.cn, identifier ChiCTR-OPC-16009113 and ChiCTR1800018137.

## Introduction

Extramedullary multiple myeloma (EMM), one of the natural courses of advanced multiple myeloma (MM), is an aggressive sub-entity. It is characterized by the involvement of multiple organs such as the central nervous system, liver, pleura, lymphatic system, skin, etc. (1, 2). EMM may be found in newly diagnosed MM or at the time of relapse (secondary EMM). Plasma cell leukemia (PCL), characterized by drug resistance, rapid progression, and short survival, is classified as a variant of aggressive EMM (3). With the development of imaging technology and the prolonged lifespan by new drugs, the diagnostic rate of EMM is increasing (4). At the disease progression stage, the incidence of EMM ranges from 10% to 30% (5, 6). Novel drugs such as monoclonal antibodies (mAbs), immunomodulatory drugs (IMiD), and proteasome inhibitors (PI) have improved the survival of MM patients. However, EMM, including PCL patients, have limited benefits from the existing strategies (3, 7–10).

Anti-BCMA CAR-T therapy achieved the most prominent responses in RRMM, with a high objective response rate (ORR) (11–17). We reviewed the published clinical trials with raw data available and found that several of these studies have enrolled EMM patients, but no analysis was performed on this specific subgroup (10, 17).Therefore, we firstly reported the differences in clinical response, adverse events, and pharmacokinetics between EMM and non-EMM patients receiving anti-BCMA CAR-T cell therapy in our center.

## Materials and Methods

### Study Conduct and Patients

**Figure 1:**
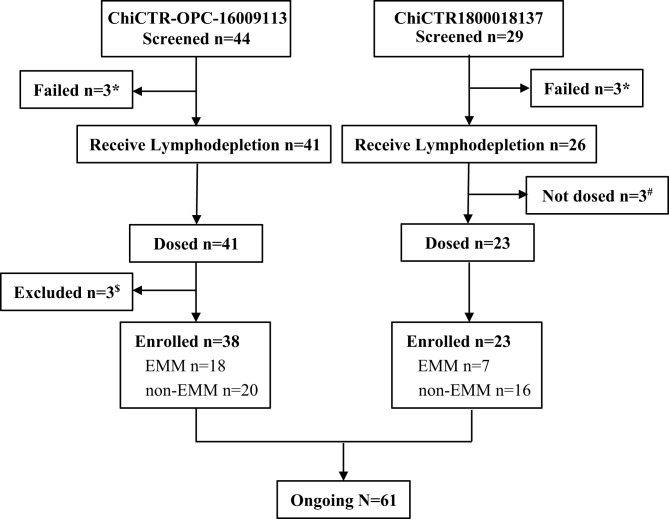
Flowchart. *Three patients failed in the screening of trail ChiCTR-OPC-16009113, and three patients failed in screening ChiCTR1800018137, respectively, because of not meet inclusion criteria or rapid progression. ^#^Three patients who received lymphodepletion were not dosed because of heart failure, severe liver function damage, and severe infection, respectively. ^$^A patient who had POEMS rather than MM or EMM was excluded, and two patients were excluded because of early death.

We reviewed and summarized the results from published anti-BCMA CAR-T clinical trials in which raw data of EMM patients was available. We then focused on the two trials conducted in our clinical centers. The phase I study of murine anti-BCMA CAR-T cell therapy was registered at Chinese Clinical Trial Registry as ChiCTR-OPC-16009113, and the phase II study of a fully human anti-BCMA CAR (CT103A) was registered at Chinese Clinical Trial Registry as ChiCTR1800018137. The murine anti-BCMA CAR product was composed of a murine anti-BCMA single-chain variable fragment (scFv), a CD8a hinge, the CD28 co-stimulatory domain (including CD28 transmembrane, and intracellular domains), and the CD3ζ activation domain. The fully human anti-BCMA CAR product (CT103A) was composed of a fully human scFv, a CD8a hinge, and transmembrane domain, 4-1BB co-stimulatory, and CD3ζ activation domains. Between February 22, 2017, and September 26, 2019, a total of 73 (murine 44; fully human 29) consecutive adult subjects with BCMA positive RRMM were screened according to the study protocols, and 12 (murine 6; fully human 6) patients were excluded (**Figure 1**).

### Assessments Criteria

Most studies agree that EM could be divided into two groups: the first group comprises tumors that are extending directly from osteolytic bone lesions (EM-B, extramedullary-bone related), while the second results from plasma cell infiltration into soft tissues, with no relationship to the bone (EM-E, extramedullary-extraosseous) (7, 8). The EMM in our study included EM-E and PCL. The PCL in our research included primary PCL and secondary PCL (7). Cytokine release syndrome (CRS) and symptoms of immune effector cell-associated neurotoxicity syndrome (ICANS) were graded according to the criteria of Lee et al. (18, 19). All other adverse events (AEs) and severe adverse events (SAEs) are evaluated by the National Cancer Institute Common Terminology Criteria for Adverse Events (CTCAE) Version 5.0 (20, 21). The diagnose, clinical response, and disease progression was assessed according to the IMWG consensus criteria at serial time points after CAR-T infusion. The data-cutoff date was April 1, 2021. CAR transgene copies in the patients’ peripheral blood monocytes were monitored by digital droplet polymerase chain reaction (ddPCR).

### Study Approval

These study protocols were approved by the institutional review board of Tongji Hospital, Tongji Medical College, Huazhong University of Science and Technology. Details of the protocols, CAR-T cell preparation, and assessments criteria were as described in our previous studies (12, 14). Both trials were conducted in compliance with the Declaration of Helsinki. Written informed consent was obtained from each participant.

### Statistical Analysis

Continuous variables were described using median and range. Categorical variables were reported in number and percentage. The analysis of categorical variables was performed by the the chi-square test, Fisher’s exact chi-square test, or Pearson’s chi squared test. For continuous variables, Wilcoxon rank-sum test was used. Kaplan-Meier method was employed to estimate the probabilities of overall survival (OS) and progression-free survival (PFS). Estimations of risk were performed by Cox regression. Statistical analyses were performed by SPSS 22 and Graphpad Prism 8. P values less than 0.05 (two-tailed) were considered statistically significant.

## Results

### Anti-BCMA CAR-T Clinical Trials and Patient Characteristics

Eight anti-BCMA CAR-T clinical trials had enrolled EMM patients (10–17,) and seven of them presented the preliminary efficacy of EMM patients. We summarized their results in **Table 1**. The ORR of EMM ranged from 57% to 100%, with the complete remission (CR) rate of 29% to 60%.

**Table 1:** Anti-BCMA CAR-T cell products in clinical trials included EMM patients.

No. of EMMpatients	Name	Clinical trial information	Major response	Reference
1 (16)	Anti-BCMA CAR-T cell therapy	NCT02215967	the ORR of EMM is 100% (1/1), with 1 (100%) VGPR (at 51 weeks); the ORR of non-EMM is 80% (12/15) with 2 (13%) sCR/CR and 9 (60%) ≥VGPR.	Brudno, J N et al. (13)
9 (33)	bb2121	Phase Ib NCT02658929	the ORR of EMM is 89% (8/9), with 4 (44%) sCR/CR and 6 (66%) ≥ VGPR; ORR of non-EMM is 83% (20/24) with 11 (33%) sCR/CR and 18 (75%) ≥VGPR.	Raje, Noopur et al. (15)
5 (17)	LCAR-B38M	Phase I/II NCT03090659	the ORR of EMM is 80% (4/5), with 3 (60%) sCR/CR and 4 (80%)≥ VGPR; ORR of non-EMM is 92% (11/12) with 9 (75%) sCR/CR and 11 (92%) ≥VGPR.	Xu, Jie et al. (11)
7 (25)	Anti-BCMA CAR-T cell therapy	Phase I NCT02546167	the ORR of EMM is 57% (4/7), with 2 (29%) sCR/CR and 4 (57%)≥ VGPR.	Cohen, Adam D et al. (16)
50 (128)	ide-cel (bb2121)	Phase II NCT03361748	CR rate: non-EMM>EMM (no accurate values); ORR: non-EMM>EMM (no accurate values).	Munshi, N C et al. (10)
7 (23)	CT103A	Phase I ChiCTR1800018137	the ORR of EMM was 100% (7/7), with 2 (29%) sCR/CR and 5 (71%) ≥ VGPR; the ORR of non-EMM was 100% (16/16), with 13 (81%) sCR/CR and 14 (88%) ≥ VGPR.	Wang, D et al. (14)
18 (38)	Murine BCMA CAR-T cell therapy	Phase I ChiCTR-OPC-16009113	the ORR of EMM was 77.78% (14/18), with 7 (39%) sCR/CR and 10 (56%) ≥ VGPR; the ORR of non-EMM was 90% (18/20), with 9 (45%) sCR/CR and 11 (55%) ≥ VGPR.	Li, C et al. (12)

EMM, extramedullary myeloma; BCMA, B-cell maturation antigen; CAR, chimeric antigen receptor; ORR, objective response rate; OS, overall survival; sCR, stringent complete response; VGPR, very good partial response.

We compared the baseline characteristics of 25 EMM patients and 36 non-EMM patients in the two trials conducted in our clinical centers (**Table 2**). The analysis showed no statistical differences between EMM and non-EMM patients in baseline characteristics except for high-risk cytogenetics. The median age of EMM and non-EMM patients was 55 (range, 34 - 70) years and 53 (range, 34 - 69) years, respectively. The median time from diagnosis to infusion was 3.2 (range, 0.8 - 12.6) years for EMM patients, and 2.9 (range, 0.7 - 11.3) years for non-EMM patients. 6 (24%) EMM patients and 18 (50%) non-EMM patients had high-risk cytogenetic profile.

**Table 2:** Comparison of baseline characteristics between EMM patients and non-EMM patients in the two clinical trials conducted in our center.

Characteristics	EMM (n = 25)	non-EMM (n = 36)	p-Value
**Age, yr, median (range)**	55 (34 - 70)	53 (34 - 69)	0.509
**Sex, n (%)**			0.353
Male	13 (52.0)	23 (63.9)	
Female	12 (48.0)	13 (36.1)	
**ECOG performance-status score**			1.000
0-1	23 (92.0)	34 (94.4)	
2-3	2 (8.0)	2 (5.6)	
**Time since diagnosis, yr, median (range)**	3.2 (0.8 - 12.6)	2.9 (0.7 - 11.3)	0.363
**Prior lines of therapy, median (range)**	4 (3 - 11)	4 (3 - 10)	0.114
**Durie-Salmon stage, n (%)**			0.145
I	1 (4.0)	0	
II	0	3 (8.3)	
III	22 (95.0)	33 (91.7)	
**ISS stage, n (%)**			0.288
I	7 (30.0)	16 (44.4)	
II	11 (48.0)	10 (27.8)	
III	5 (22.0)	10 (27.8)	
**Myeloma type, n (%)**			0.104
IgG κ	5 (20.0)	8 (22.2)	
IgG λ	9 (36.0)	10 (27.8)	
IgA κ	2 (8.0)	3 (8.3)	
IgA λ	0	2 (5.6)	
IgD λ	1 (4.0)	3 (8.3)	
Light chain κ	0	6 (16.7)	
Light chain λ	7 (28.0)	2 (5.6)	
Non-secretor	1 (4.0)	2 (5.6)	
**High risk cytogenetics, n (%)^&^**	6 (24.0)	18 (50.0)	0.041
**TP 53 mutations, n (%)**	3 (13.6)	3 (9.1)	0.674
**BCMA MFI on plasma cells, median (range)**	2417 (840 - 12516)	1586 (303 - 51023)	0.133
**CAR-T structure, n (%)**			0.192
Murine	18 (72.0)	20 (55.6)	
Fully human	7 (28.0)	16 (44.4)	

EMM, extramedullary myeloma; BCMA, B-cell maturation antigen; CAR, chimeric antigen receptor; ECOG, Eastern Cooperative Oncology Group; DS, Durie Salmon; ISS, International Staging System; MFI, Mean Fluorescence Intensity.

^&^Cytogenetic features were measured using florescence in situ hybridization. The probes include t(4;14), Del(17p), 1q21, t(14;16), Del (13q), t(11;14) and. High risk cytogenetic features (any t(4;14), Del(17p), and t(14;16)) evaluated with conventional cytogenetics or fluorescence in-situ hybridization (FISH).

### Safety

**Figure 2:**
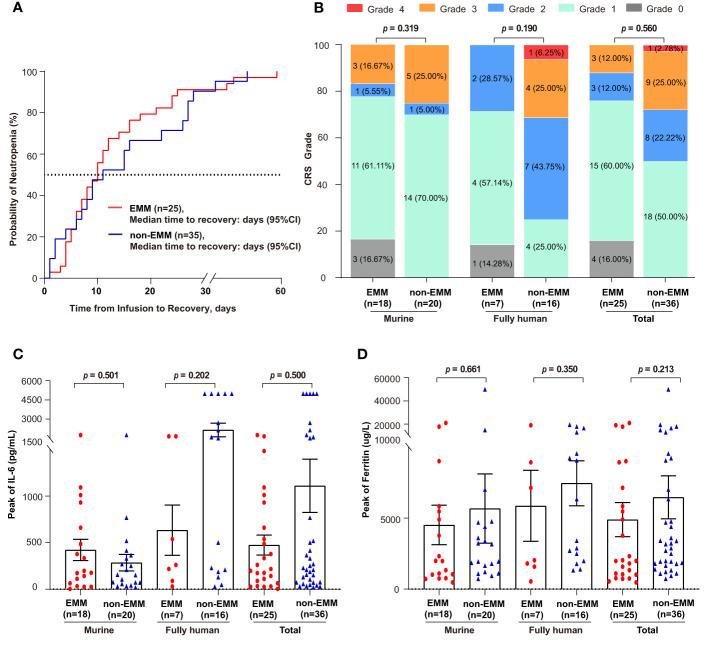
Comparison of time to recovery of neutrophil, cytokine release syndrome (CRS), and inflammatory factors between EMM patients and non-EMM patients receiving anti-BCMA CAR-T cell therapy in two clinical trials conducted in our center **(A)** Time to the recovery of patients with grade 3/4 neutropenia is shown. One non-EMM patient was excluded because the neutrophil count had not recovered until death (OS = 20 days). Time to recovery, which is defined as absolute neutrophil count ≥1000 cells/μL from infusion till the first time of recovery. **(B–D)** Comparison of the CRS grade, the peak levels of IL-6 and Ferritin.

A total of 73 different types of adverse events were recorded, and those with incidence ≥ 10% were summarized in **Table 3**. No statistical differences were observed between EMM and non-EMM patients in all AEs. The most common AEs of grade ≥ 3 in both groups was hematologic toxicities, including leukopenia, lymphopenia, neutropenia, anemia, and thrombocytopenia. The median recovery times of neutropenia for EMM and non-EMM patients were 9 (range, 0 to 45) days and 10 (range, 0 to 58) days post-infusion, respectively (**Figure 2A**). Delayed recovery of neutropenia (4.00% in EMM and 8.57% in non-EMM) was observed in both groups.

**Table 3:** Comparison of adverse events occurred in 10% or more patients during the first eight weeks post-infusion between EMM and non-EMM patients in two clinical trials conducted in our center.

	EMM (n = 25)				non-EMM (n = 36)				*p*-Value
Adverse event	Grade 1-2n (%)	Grade 3n (%)	Grade 4n (%)	Any Graden (%)	Grade 1-2n (%)	Grade 3n (%)	Grade 4n (%)	Any Graden (%)	
**Hematologic**									
Leukopenia	1 (4.0)	1 (4.0)	23 (92.0)	25 (100.0)	1 (2.8)	1 (2.8)	34 (94.4)	36 (100.0)	1.000
Neutropenia	1 (4.0)	2 (8.0)	22 (88.0)	25 (100.0)	0 (0.0)	1 (2.8)	35 (97.2)	36 (100.0)	0.410
Lymphopenia	0 (0.0)	0 (0.0)	25 (100.0)	25 (100.0)	0 (0.0)	0 (0.0)	36 (100.0)	36 (100.0)	–
Anemia	6 (24.0)	15 (60.0)	2 (8.0)	23 (92.0)	4 (11.1)	28 (77.8)	4 (11.1)	36 (100.0)	0.056
Thrombocytopenia	6 (24.0)	3 (12.0)	16 (64.0)	25 (100.0)	4 (11.1)	3 (8.3)	29 (80.6)	36 (100.0)	0.292
**Coagulative**									
Prolonged APTT	17 (68.0)	0 (0.0)	0 (0.0)	17 (68.0)	28 (77.8)	1 (2.8)	0 (0.0)	29 (80.6)	1.000
Fibrogenopenia	4 (16.0)	1 (4.0)	0 (0.0)	5 (20.0)	15 (41.7)	2 (5.6)	0 (0.0)	17 (47.2)	1.000
**Metabolic**									
Hypokalemia	18 (72.0)	0 (0.0)	0 (0.0)	18 (72.0)	19 (52.8)	3 (8.3)	0 (0.0)	22 (61.1)	0.262
Hyponatremia	8 (32.0)	2 (8.0)	0 (0.0)	10 (40.0)	12 (33.3)	1 (2.8)	1 (2.8)	14 (38.9)	1.000
Hypocalcemia	14 (56.0)	1 (4.0)	0 (0.0)	15 (60.0)	20 (55.6)	0 (0.0)	0 (0.0)	20 (55.6)	0.410
Elevated ALT	7 (28.0)	2 (8.0)	0 (0.0)	9 (36.0)	11 (30.6)	1 (2.8)	0 (0.0)	12 (33.3)	0.562
Elevated AST	6 (24.0)	2 (8.0)	0 (0.0)	8 (32.0)	13 (36.1)	3 (8.3)	0 (0.0)	16 (50.0)	1.000
Heart failure	4 (16.0)	0 (0.0)	2 (8.0)	6 (24.0)	8 (22.2)	0 (0.0)	1 (2.8)	9 (25.0)	0.562
Arrhythmia	5 (20.0)	3 (12.0)	0 (0.0)	8 (32.0)	9 (25.0)	0 (0.0)	0 (0.0)	9 (25.0)	0.064
Creatinine increased	3 (12.0)	2 (8.0)	0 (0.0)	5 (20.0)	3 (8.3)	1 (2.8)	0 (0.0)	4 (11.1)	0.562
**Others**									
Fever	16 (64.0)	3 (12.0)	0 (0.0)	19 (76.0)	26 (72.2)	6 (16.7)	0 (0.0)	32 (88.9)	0.725
Lung infection	0 (0.0)	9 (36.0)	0 (0.0)	9 (36.0)	3 (8.3)	17 (47.2)	1 (2.8)	21 (58.3)	0.307
Upper respiratory infection	3 (12.0)	1 (4.0)	0 (0.0)	4 (16.0)	3 (8.3)	0 (0.0)	0 (0.0)	3 (8.3)	0.410

APTT, activated partial thrombin time; ALT, alanine aminotransferase; AST, aspartate aminotransferase. The grading of AE was according to the CTCAE 4.03. The P value is based on Fisher’s exact test, or Pearson’s chi-squared test. P values less than 0.05 (two-tailed) were considered statistically significant.

Patients with EMM tended to have lower cytokine release syndrome (CRS) grade than individuals without EMD in both trials, although the difference was not statistically significant (**Figure 2B**). The incidence of ≥ grade 3 CRS was 12% in EMM patients and 27.78% in non-EMM patients, respectively. Only one of the non-EMM patients that received murine anti-BCMA CAR-T therapy experienced ICANS. No differences were observed in serum ferritin and IL-6 levels between the two groups (**Figures 2C** **, D**).

### Efficacy

**Figure 3:**
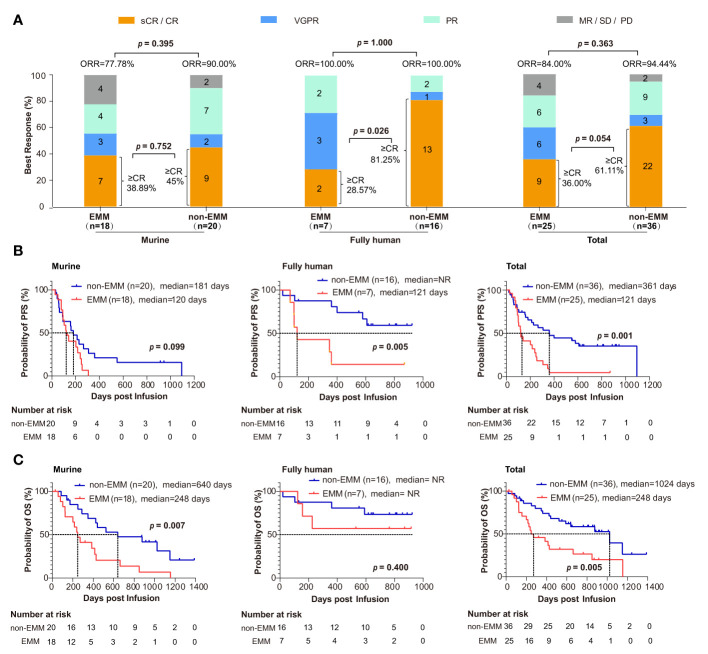
Comparison of clinical response and survival analysis between EMM patients and non-EMM patients receiving anti-BCMA CART cell therapy in two clinical trials conducted in our center **(A)** ORR was analyzed between EMM and non-EMM. The *p* value is based on Fisher’s exact test. **(B, C)** Analysis of PFS and OS for EMM and non-EMM patients using the Kaplan-Meier method. P values less than 0.05 (two-tailed) were considered statistically significant. CR, complete response; EMM, extramedullary myeloma; ORR, objective response rate; MR, minimal response; NR, not reach; OS, overall survival; PD, relapse/progressive disease. PFS, progression-free survival; PR, partial response; sCR, stringent complete response; SD, stable disease; VGPR, very good partial response.

There was no significant difference in the ORR and ≥ CR rate between EMM and non-EMM patients. The ORR of EMM and non-EMM patients was 84.00% (21/25) and 94.44% (34/36), respectively (**Figure 3A**; *p* = 0.363). The ≥ CR rate of EMM and non-EMM patients was 36.00% (9/25) and 61.11% (22/36), respectively (**Figure 3A**; *p* = 0.054). However, for patients receiving the fully human anti-BCMA CAR-T cell therapy, the ≥ CR rate of EMM patients was lower than non-EMM patients (**Figure 3A**; 28.57% vs. 81.25%; *p* = 0.026).

The median follow-up time was 873 days. The Kaplan-Meier method showed that there were significant differences in PFS (121 days vs. 361 days, *p* = 0.001) and OS (248 days and 1024 days, *p* = 0.005) for all the EMM and non-EMM patients (**Figures 3B** **, C**). Interestingly, for the patients that received murine CAR, the difference was only observed in OS (248 vs. 640 days, *p* = 0.007) other than PFS (120 vs. 181 days, *p* = 0.099). While for patients that received fully human CAR, the difference was only observed in PFS (121 days vs. not reach (NR); *p* = 0.005) other than OS (both NR; *p* = 0.400). The rates of OS for EMM and non-EMM patients that received fully human CAR were 57.14% (4/7) and 75% (12/16) at one year, respectively.

### Pharmacokinetics

**Figure 4:**
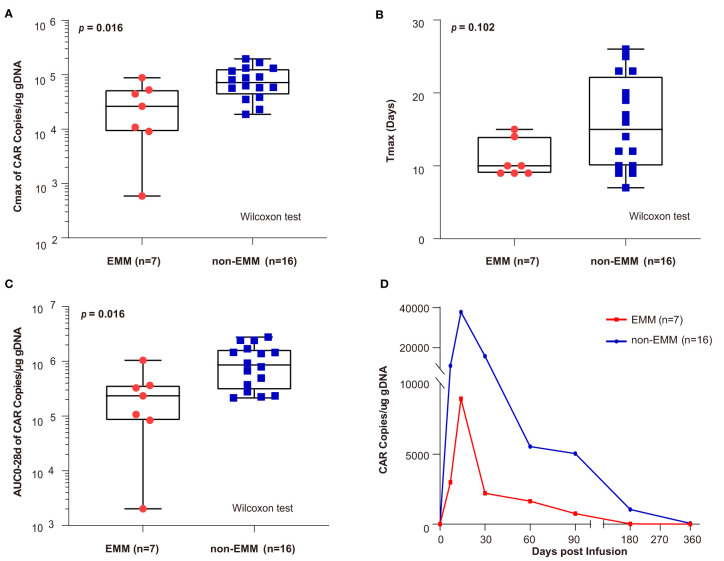
Comparison of CAR transgene kinetics between EMM patients and non-EMM patients receiving novel fully human anti-BCMA CAR-T infusion conducted in our center **(A–C)** the comparison of C_max_, T_max_, and area under the curve of the transgene level from infusion to 28 days (AUC_0–28d_) between EMM and non-EMM patients that received novel fully human anti-BCMA CAR-T therapy. The *p* value is based on a two­sided Wilcoxon rank­sum test and *p* values less than 0.05 (two-tailed) was considered statistically significant. **(D)** The Car copies from infusion to the last follow-up were compared between EMM and non-EMM patients. Data presented as a geometric mean. AUC_0­28d_, area under the curve during the first 28 days after the infusion; Cmax, peak value of CAR copies; Tmax, the time elapsed till peak value appeared.

After infusion, the peak value of CAR copies (C_max_) and the area under the curve of the transgene level from infusion to 28 days (AUC_0–28d_) in EMM patients was lower than in non-EMM patients (C_max_, *p* = 0.016; AUC_0–28d_, *p* = 0.016) (**Figures 4A, C**). There was no difference in the T_max_ for EMM and non-EMM patients (**Figure 4B**). As shown in **Figure 4D**, the CAR copies of EMM patients were lower than non-EMM patients from the infusion to the last follow-up. CAR-T cells tended to have lower expansion in EMM patients than in non-EMM patients.

### Risk Factors

**Figure 5:**
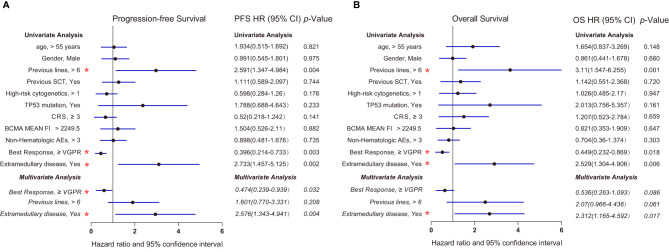
Forest plots showing the association between risk factors and survival in our center **(A)** Progression-free survival (Cox regression, HR, and 95% CI). **(B)** Overall survival (Cox regression, HR, and 95% CI). Multivariate Cox regression analysis was performed by using the significant prognostic factors identified in univariate analysis. Multivariate analysis was indicated in italics. *p* values less than 0.05 (two-tailed) were considered statistically significant (highlighted with red “*”). AEs, adverse events; CRS, cytokine release syndrome; HR, hazard ratio; MFI, Mean Fluorescence Intensity; SCT, stem cell transplantation; VGPR, very good partial response.

We further analyzed the factors that may impact the OS and PFS of patients receiving anti-BCMA CAR-T therapy in a Cox model (**Figure 5**). Univariate Cox regression analysis showed that previous lines, best response, and extramedullary diseases were significantly associated with OS and PFS (p < 0.05). Multivariate Cox regression analysis revealed that extramedullary disease was also an independent prognostic risk factor in RRMM patients receiving anti-BCMA CAR-T cell therapy (hazard ratio, 2.576; 95% CI, 1.343 to 4.941; *p* = 0.004; OS hazard ratio, 2.312; 95% CI, 1.165 to 4.592; *p* = 0.017).

## Discussion

In general, the prognosis of EMM, including PCL patients, is poor. There is currently no consensus on the standard regimen for EMM patients, and few clinical studies are designed for them. The efficacy of conventional chemotherapeutic drugs and novel agents, either alone or combined, was limited in EMM patients (7, 8, 22). The ORR of secondary EMM patients receiving novel agents, such as Carfilzomib, Daratumumab, Lenalidomide, etc., was mostly reported no more than 50% (23–26). What’s more, the median PFS and OS of daratumumab-based therapy for EMM patients were only 69 days and 198 days, respectively, in one study (26). In another study that included 357 MM (24 secondary EMM) patients, the median PFS and OS for these secondary EMM patients was about two months and seven months, respectively (27).

Compared to the drugs’ limited effect mentioned above, anti-BCMA CAR-T therapy is a promising strategy for RRMM patients with EMM (10–16). In our study, the ORR of EMM patients reached 100% with ≥ CR rate of 28.57% in the fully human trial, which was significantly higher than existing regimens, and the median PFS and OS were also longer. Similar results were reported by other studies (28–32). These encouraging results showed that anti-BCMA CAR-T therapy has obvious predominance over the existing drugs in response rate, depth of remission, and survival. But in subgroup analysis, the treatment efficacy in EMM patients was not as satisfactory as in non-EMM patients, as we observed lower ≥ CR rate and shorter PFS/OS in EMM patients. As we analyzed the murine and fully human CAR separately, the difference between EMM and non-EMM patients was only observed in PFS for patients receiving fully human CAR. Meanwhile the difference in OS was only observed in patients receiving murine CAR. These findings may result from the advantage in remission depth of our fully human CAR over our murine CAR (12, 14), as increased depth of response is often associated with improved response durability (10). Moreover, our study demonstrated that extramedullary disease was an independent prognostic risk factor for RRMM patients receiving anti-BCMA CAR-T therapy.

Most published studies have demonstrated the safety of anti-BCMA CAR-T cell therapy to RRMM. However, there is limited data available for EMM patients (10, 17). In our study, we demonstrated that anti-BCMA CAR-T cell therapy is safe for EMM patients. In addition, we found that EMM patients tended to have a lower grade of CRS than non-EMM patients. The low expansion of CAR-T cells in EMM patients may be one of the reasons for lower CRS grade and poorer efficacy. Although ORR was over 90% in both trials, our fully human CAR has significantly longer persistency than our murine CAR (12, 14). The mechanism for the poor persistency of CAR T-cells is complicated. T-cell exhaustion and senescence, immune escape, costimulatory domain selection, generation of anti-drug antibody, and other mechanisms may contribute to the low expansion of CAR T-cells (33–36). EMM subclones are highly heterogenic, which can more easily generate clones with escape mutations of BCMA (7). Moreover, subclones of EMM could thrive and grow independent of the bone marrow microenvironment, resulting in a relatively high-risk and more ‘hostile’ microenvironment for the penetration and persistence of CAR T-cells (37). How EMM negatively impacts CAR-T efficacy is still unknown and needs further investigation.

Taken together, this work described the efficacy and safety of anti-BCMA CAR-T cell therapy in EMM patients from the two clinical trials conducted in our center. According to our studies, although it holds great promise for those patients, the duration and depth of remission seems to be limited compared with non-EMM patients. Further trials are needed to combine CAR-T cell therapy with other new agents, or stem cell transplant, to achieve a better result in EMM patients.

## Data Availability Statement

For original data, please contact the corresponding author.

## Ethics Statement 

The studies involving human participants were reviewed and approved by Tongji Hospital, Tongji Medical College, Huazhong University of Science and Technology. The patients/participants provided their written informed consent to participate in this study.

## Author Contributions

JZ, CL, and DW designed the clinical study, screened and recruited participants, examined patients and analyzed the clinical data. Moreover, they also supervised the CAR-T cell production for preclinical quality control. MX collected clinical data and was responsible for patient follow-up. YQ checked, extracted, and analyzed data and performed statistical analyses. What’s more, YQ also interpreted data and wrote the manuscript. YQ and YX edited and formatted the document. All authors contributed to the article and approved the submitted version.

## Funding

This work is mainly funded by the National Natural Science Foundation of China (81873452 and 82170223 to CL).

## Conflict of Interest

JZ is among the inventors of patent applications related to the novel fully human antiBCMA CAR-T (CT103A). JZ is a nonpaid member of Scientific and Medical Advisory Board of Nanjing IASO Therapeutics Ltd.

The remaining authors declare that the research was conducted in the absence of any commercial or financial relationships that could be construed as a potential conflict of interest.

## Publisher’s Note

All claims expressed in this article are solely those of the authors and do not necessarily represent those of their affiliated organizations, or those of the publisher, the editors and the reviewers. Any product that may be evaluated in this article, or claim that may be made by its manufacturer, is not guaranteed or endorsed by the publisher.
